# Acute polymorphic psychosis: An interesting case report

**DOI:** 10.1192/j.eurpsy.2021.2145

**Published:** 2021-08-13

**Authors:** V. Mari, A. Georgiadi, A. Maris, I. Gkolia

**Affiliations:** 1 Methexis Unit, Psychiatric Hospital of Thessaloniki, Thessaloniki, Greece; 2 3rd Short Stay Unit, Psychiatric Hospital of Thessaloniki, Thessaloniki, Greece; 3 Department Of Orthopaedics, Great Ormond Street Hospital, London, United Kingdom

**Keywords:** acute polymorphic psychosis, immediate recovery, stress factor, paliperidone

## Abstract

**Introduction:**

Acute Polymorphic Psychotic Disorder is a psychotic disorder with an acute onset, presenting thought and perception disorders variable into hours. Often, an emotional fluctuation is present and it may have a sudden onset and a rapid remission.

**Objectives:**

The review’s objective is to manifest acute polymorhic psychotic disorders and possible effective medical interventions.

**Methods:**

The current case concerns a 52-year old mother of 4 children with the manifestation of acute polymorphic psychotic disorder with a background of a stressful factor. The patient was involuntarily hospitalized in the Psychiatric Hospital of Thessaloniki from 04/01/ 2019 -21/ 01/2019 due to disorganization and acute confusing condition within the last 9 days. Delusional ideas of religious content were first observed, which alternated with ideas of greatness and then persecution, association and self-denial. She also presented auditory hallucinations while there was a fluctuation of emotion from excessive euphoria to depression. The patient had no previous hospitalization in a psychiatric clinic, however, 7 months ago she experienced another acute psychotic epeisode, while at the age of 17 and under the influence of intense stress, she described mood disorders.

**Results:**

The current symptoms subsided after one week from the day of admission. During her hospitalization, a brain CT was performed without presence of pathological findings. Initially, her medication included i.m. haloperidol 15mg / ml daily, followed by a change to per os paliperidone 9mg daily.

**Conclusions:**

Her mental status was improved, with no disturbances of consciousness noted and she was discharged on paliperidone as home medication.

**Disclosure:**

No significant relationships.

